# Wastewater-based surveillance for SARS-CoV-2 in Alberta

**DOI:** 10.23889/ijpds.v9i5.2616

**Published:** 2024-09-18

**Authors:** Danielle Southern, Casey Hubert, Michael Parkins, Tyler Williamson

**Affiliations:** 1 Centre for Health Informatics, University of Calgary; 2 University of Calgary

## Objectives and Approach

Alberta's largest research universities collaborated to expand COVID-19 wastewater monitoring throughout the province to regularly provide evidence of SARS-CoV-2 burden in municipalities representing 3.2 million people.

## Results

Sampling was conducted at 26 wastewater treatment plants and facilities across the province. The project quantified SARS-CoV-2 genomic material in wastewater to reveal population-level trends of COVID-19 cases. This inclusive and comprehensive strategy captures everyone who contributes to wastewater, including those not clinically diagnosed. Researchers collected wastewater samples in municipalities three times a week. Additional sentinel monitoring was undertaken in neighbourhoods, hospitals, long-term care facilities, worksites, shelters, and schools. Results were shared on the public COVID Data Tracker website (https://covid-tracker.chi-csm.ca/). Data was additionally linked with hospital outcomes, workforce absenteeism and outbreak information.

## Results

Wastewater-based surveillance (WBS) for SARS-CoV-2 genomic RNA associates very strongly with clinically diagnosed cases and health resource utilization, providing a ≥6-day leading indicator. WBS can effectively be performed across a range of geographic scales (from cities to individual facilities), ensuring actionable data that is relevant to end-users. We have published how outbreaks across a range of high-risk facilities can be monitored and predicted with WBS and can also be used to model COVID-19-associated workforce absenteeism. Emails and website interactions suggested widespread citizen engagement using data for evidence-based decisions.

## Conclusion

WBS is a valuable tool for identifying potential outbreaks and tailoring response measures at the policy level and by individual citizens. We've created customizable real-time data-sharing tools catering to both the public (enhanced data transparency) and government (actionable insights).

